# Introducing COSIG: The Collection of Open Science Integrity Guides

**DOI:** 10.1371/journal.pbio.3003817

**Published:** 2026-06-10

**Authors:** Yagmur Ozturk, Solal Pirelli, Reese A. K. Richardson

**Affiliations:** 1 Laboratoire d’Informatique de Grenoble, Université Grenoble Alpes, Grenoble, France; 2 Programming Methods Laboratory, École Polytechnique Fédérale de Lausanne, Lausanne, Switzerland; 3 Center for Science of Science and Innovation, Kellogg School of Management, Northwestern University, Evanston, Illinois, United States of America

## Abstract

Investigating the integrity of published scientific papers is key to the scientific process, but the necessary knowledge is in short supply. This Community Page presents COSIG, an open collection of meta-scientific guides enabling anyone to perform forensic peer review.

## Teaser

Investigating the integrity of published scientific papers is key to the scientific process, but the necessary knowledge is in short supply. We present COSIG, an open collection of meta-scientific guides enabling anyone to perform forensic peer review.

## Introduction

The reliability of the scientific literature is under increasing scrutiny. Both institutional and external observers agree that research misconduct poses a major threat [1]. This is in part due to the rise of paper mills [2], organizations that sell fabricated articles that typically pass basic scrutiny but fall apart upon closer inspection. Such inspection requires expert knowledge and skills from a specific domain, such as detecting inconsistent statistics or nonfunctional nucleotide sequence reagents. Having the expertise necessary to detect problematic scientific articles is not a given for all scientists, nor for all research integrity officers.

Currently, the main way for experts to call attention to publication integrity issues for preprints and published articles, including potential research misconduct, is through some form of post-publication peer review (PPPR). This process involves leaving public comments on papers, often in a similar format to pre-publication review, although in this case, authors are not required to respond. This form of PPPR often occurs on the PPPR platform PubPeer owing to the platform’s ease of use and support for authenticated and anonymous comments, as well as for author responses.

Articles might also be screened for potential integrity issues before and during traditional, pre-publication peer review. These tasks are very similar to integrity-focused PPPR and thus much of the same expertise applies; for instance, the Problematic Paper Screener, a tool borne out of PPPR, has now been integrated into pre-publication editorial workflows by major publishers [3]. To recognize the generalizability of these skills across different stages of research communication, in this Community Page, we refer to all these tasks under the umbrella term ‘forensic peer review’ [4].

The current paradigm of forensic peer review is depth-based (i.e., a small number of people spend a lot of time flagging problems in their field of expertise). Because forensic peer review is not an expected part of scientists’ work and is typically not considered in evaluations of scientific impact and productivity, it is typically done as a service to science on top of existing responsibilities. This model is ill-suited to the challenge of maintaining the integrity of the literature, as there are too many problematic papers in the published literature compared to the available free time of experts.

Decontaminating the scientific literature in a more scalable way will require broadening participation in forensic peer review towards a scalable breadth-based model, wherein many people each spend a small amount of time performing forensic peer review. However, there are many barriers to participation, such as not knowing where to start, lacking domain-specific expertise, and fearing personal or professional consequences. Thus, there is a need for resources on how to perform effective forensic peer review and for greater knowledge on how to catch common publication integrity issues. These resources should be seen as authoritative by the community, providing a common language between the ‘sleuths’ who report problems and the integrity officers at publishers and research institutions who receive such reports.

## The collection of open science integrity guides

To address this problem, we present the Collection of Open Science Integrity Guides (COSIG). The goal of COSIG is to enable anyone to be a steward of the scientific literature. Using COSIG, anyone can learn how to interpret specific kinds of data, common integrity issues, and how to constructively report problems. COSIG breaks down the barriers to participating in the decontamination of the scientific literature by providing clear starting points, teaching the necessary expertise, and acting as an authoritative source that can be referred to by anyone who needs to justify their remarks when performing forensic peer review.

**Fig 1:**
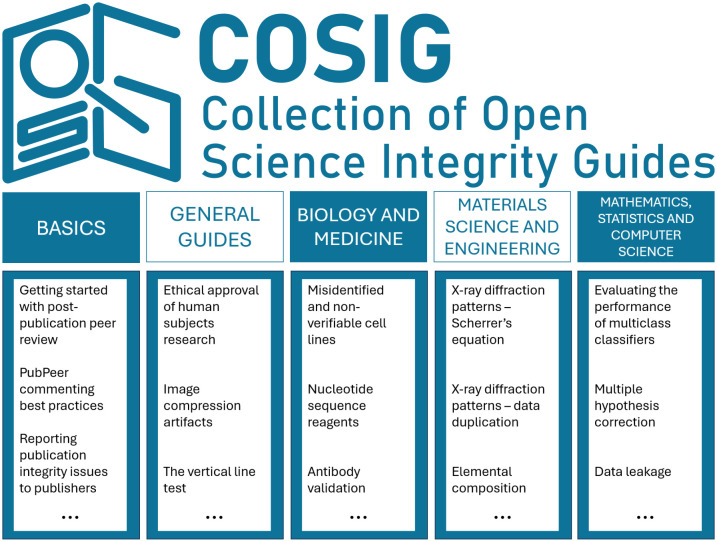
Examples of guides currently in COSIG. The Collection of Open Science Integrity Guides (COSIG) currently consists of 35 guides but is constantly expanding.

COSIG currently consists of 35 guides written by 22 experts, including guides on general subjects (e.g., PubPeer commenting guidelines), specific subjects (e.g., interpreting Tauc plots in electrochemical studies), and methods (e.g., extracting data points from vector graphics; see Fig 1 for more examples).

## Using COSIG

COSIG is suitable for both new and experienced post-publication reviewers and traditional peer reviewers, as well as research integrity officers, editors, and educators. Post-publication reviewers can use COSIG to learn the basics if they are new to forensic peer review and learn from expert-written guides on many subjects. COSIG guides can be cited when writing comments, thus strengthening the reviewer’s confidence in the validity of their concerns. Traditional peer reviewers can also use COSIG to this effect when communicating concerns to editors and authors pre-publication.

Editors and integrity officers at publishers can use COSIG when investigating the reliability of papers under criticism and can integrate COSIG guides into training materials and use them when screening submissions for potential issues. While many publishers already employ dedicated staff with expertise in investigating individual articles and large-scale manipulation of the publication process, openly accessible resources such as COSIG can support these efforts by providing shared guidance that can be used across publishers, institutions, and the wider research community.

Integrity officers at research institutions can use COSIG to speed up misconduct investigations. Indeed, when reports are received that cite COSIG guidance, research integrity officers may be able to proceed without needing to engage outside experts. This is particularly important when dealing with large-scale misconduct investigations, as there can be tens or even hundreds of affected papers.

Educators can use COSIG as the foundation for teaching aspiring scientists and academics on how to spot problems when reading papers. Indeed, assessing scientific claims, spotting potential weaknesses, checking the provenance of claims and articulating critiques are essential skills for all scientists. COSIG represents an open and adaptable resource for educators to develop teaching materials to foster these skills.

Finally, anyone can develop new detection tools based on material covered in COSIG. Some guides focus on problems that might be amenable to automated or semi-automated detection, pre- or post-publication. COSIG provides foundational materials for anyone with relevant skills in writing and applying these tools, which could prove useful for improving the throughput and timeliness of detection.

## Limitations of COSIG

COSIG mainly addresses epistemic barriers to engaging in forensic peer review by providing guides on how to identify and communicate potential issues. However, participation in forensic peer review is influenced by further factors, including organizational and structural barriers such as fear of professional repercussions, time limitations, or lack of institutional support.

Additionally, in certain cases, forensic peer review can require substantial domain expertise beyond that which COSIG can provide. COSIG should therefore be considered a resource to support skill development and documenting of potential concerns, rather than as a solution to all barriers to forensic peer review. Finally, although COSIG can help introduce newcomers to forensic peer review, it does not supplant the role of practice and experience in becoming an effective forensic peer reviewer.

## Invitation to contribute and future plans

We encourage everyone to contribute to COSIG, in the form of improving an existing guide or writing a new guide. We are especially looking for new guides concerning social and behavioral sciences (e.g., psychology). We plan to keep growing COSIG by finding new contributors and we hope to translate the core guides into languages other than English for greater global reach.

As an open-source project, COSIG’s materials are publicly available and anyone can propose improvements or new guides. However, all contributions are reviewed and curated by the COSIG maintainers, and nothing is automatically updated in the official version without editorial oversight. We work with contributors to improve the pedagogical aspects, and we vet the scientific aspects by asking experts in relevant fields to review contributions, ensuring that COSIG features high-quality, adaptable material.
